# The Validity of Peer Review in a General Medicine Journal

**DOI:** 10.1371/journal.pone.0022475

**Published:** 2011-07-25

**Authors:** Jeffrey L. Jackson, Malathi Srinivasan, Joanna Rea, Kathlyn E. Fletcher, Richard L. Kravitz

**Affiliations:** 1 Division of General Medicine, Zablocki VA Medical Center, Milwaukee, Wisconsin, United States of America; 2 Division of General Medicine, University of California Davis, Sacramento, California, United States of America; Johns Hopkins University, United States of America

## Abstract

All the opinions in this article are those of the authors and should not be construed to reflect, in any way, those of the Department of Veterans Affairs.

**Background:**

Our study purpose was to assess the predictive validity of reviewer quality ratings and editorial decisions in a general medicine journal.

**Methods:**

Submissions to the Journal of General Internal Medicine (JGIM) between July 2004 and June 2005 were included. We abstracted JGIM peer review quality ratings, verified the publication status of all articles and calculated an impact factor for published articles (Rw) by dividing the 3-year citation rate by the average for this group of papers; an Rw>1 indicates a greater than average impact.

**Results:**

Of 507 submissions, 128 (25%) were published in JGIM, 331 rejected (128 with review) and 48 were either not resubmitted after revision was requested or were withdrawn by the author. Of 331 rejections, 243 were published elsewhere. Articles published in JGIM had a higher citation rate than those published elsewhere (Rw: 1.6 vs. 1.1, p = 0.002). Reviewer quality ratings of article quality had good internal consistency and reviewer recommendations markedly influenced publication decisions. There was no quality rating cutpoint that accurately distinguished high from low impact articles. There was a stepwise increase in Rw for articles rejected without review, rejected after review or accepted by JGIM (Rw 0.60 vs. 0.87 vs. 1.56, p<0.0005). However, there was low agreement between reviewers for quality ratings and publication recommendations. The editorial publication decision accurately discriminated high and low impact articles in 68% of submissions. We found evidence of better accuracy with a greater number of reviewers.

**Conclusions:**

The peer review process largely succeeds in selecting high impact articles and dispatching lower impact ones, but the process is far from perfect. While the inter-rater reliability between individual reviewers is low, the accuracy of sorting is improved with a greater number of reviewers.

## Introduction

Nearly all scientific journals rely on peer review to make decisions about publishing submitted manuscripts. Peer review, in which external experts critique manuscripts being considered for publication by journals, is believed to serve two purposes: improving the quality of manuscripts and selecting higher value articles. Manuscript quality has been found to improve after peer review [1]–[4] and authors believe peer review improves their manuscripts [5], [6]. Other studies have shown that editors are strongly influenced by reviewer recommendations, 3 and that editor perception of reviewer quality varies greatly [6]. Stephen Lock first discussed the peer review process in his book ‘A Difficult Balance’ pointing out potential problems with the scientific peer review process [7]. Subsequently several studies have cast doubt on the reliability of peer review, finding that the rate of agreement between reviewers is low [8]–[11].There are scant data on whether reviews help discriminate high from low value articles. In one study that rated the quality of reviews, there was little correlation between the ratings of the quality of the reviews given by the editor and whether or not the editor accepted the reviewers' recommendation regarding publication [6]. Another study looked at the relationship between reviewer ratings and the subsequent number of citations for published articles in a non-medicine scientific journal (Angewandte Chemie International Edition), [12], [13] finding that the review process accurately distinguished articles with high and low impact as assessed by the number of subsequent citations. However, this has not, to our knowledge, been examined in any medical journal. The authors of a recent Cochrane review on the value of peer review for biomedical journals concluded, “at present, little empirical evidence is available to support the use of editorial peer review as a mechanism to ensure quality of biomedical research. [14]”

Our study's purpose was to evaluate the predictive validity of the peer-review process at the Journal of General Internal Medicine, a journal for academic generalists featuring articles on primary care, hospital practice, clinical epidemiology, health services research and policy, and medical education. Specifically, this study examines the impact of original research manuscripts both published and rejected by JGIM based on peer review ratings, using subsequent manuscript publication and citation number as measures of impact. We hypothesized that articles rejected by JGIM and published in other journals would have a lower rate of citations than those accepted by JGIM. We secondarily hypothesized that the rating of the quality of the articles by reviewers would correlate with article citation rates.

## Methods

### Articles

All articles submitted to JGIM between 1 July 2004 and 30 June 2005 as original research or systematic review articles were included in this analysis. We excluded submissions in response to calls for supplements, as this is a special population of articles that has a different review process and acceptance priorities. This time period was selected to allow articles not accepted by JGIM sufficient time to be published and cited by other journals. We determined whether the articles rejected by JGIM were subsequently published in another journal by searching PUBMED and GOOGLE ™ using the title of the article and the author's names. For articles not located in PUBMED or GOOGLE™, we contacted the authors by email and asked if the article had been published and for the article citation. This protocol was approved by the IRB at the Zablocki VA Medical Center. We received no funding to complete this project.

### JGIM Review Process

During the time frame for this study, JGIM had 2 editors and 30–40 deputy editors. All submitted articles are initially reviewed by one of the two editors; articles may be rejected at this level without further review, though generally only articles that are deemed inappropriate for the journal readership or extremely poorly written are rejected at this stage. Articles passing this screen are assigned to a deputy editor with expertise in the topic area. Deputy editors perform a more careful reading and can decide to reject the article without further review or send it out for external review. Once external reviewers' comments return, the associate editor may accept the paper as is, request a revision from the authors, or reject the paper. If revisions are requested, the revised and resubmitted manuscript is returned to the deputy editor for final adjudication (accept, revise further, or reject). As a general rule, revised articles are not sent back out for additional external review.

### Article Review

Manuscripts submitted to JGIM and sent for external review are rated by reviewers on six quality domains. Five (interest to JGIM readership, originality, statistical analysis, validity of conclusions and clarity of writing) are rated on a five point scale. The sixth quality domain, study design, is rated on a three point scale (acceptable, minor flaws, major flaws). From these reviewer ratings of manuscript quality, we calculated an average quality rating of each manuscript for each reviewer by summing the scores assigned in each of the 6 domains and dividing by 6. In addition to rating quality, reviewers are asked to make a recommendation regarding publication. They can recommend that the manuscript be “accepted as is”, “conditionally accepted”, “reconsidered with minor or major revisions”, or “rejected.”

### Article Importance

We used as our measure of article importance the number of times it was cited by other authors over the three years immediately following publication. While citation rates are an imperfect measure of the importance and quality of an article, the Cochrane collaboration identified article citation rates as a good surrogate marker for both the importance and the relevance of biomedical articles [13]. The frequency of citations for published articles was abstracted from the Science Citation Index for up to six years after the publication date. Unpublished articles were given a citation rating of 0 for all six years. To determine the relative impact for each published article, an Rw 15 was calculated by summing the number of citations for each article for the 3 calendar years immediately after publication and dividing it by the average number of citations for this cohort of articles. In order to give all articles the opportunity to have a 3-year citation window, we excluded articles that were published later than 2007. An Rw of greater than 1.0 indicates that the article had greater than average impact; articles with an Rw less than 1.0 had less than average impact than the articles in this study. In this study there are several possible outcomes: 1) articles can be rejected by JGIM and not published elsewhere, 2) articles can be rejected by JGIM and published elsewhere and have an Rw higher or lower than 1.0 or 3) articles can be published in JGIM and have either a higher or lower Rw than 1.0. From a journal's perspective, desirable outcomes are that 1) accepted articles have an Rw greater than 1.0, or 2) rejected articles are either not published elsewhere or have an Rw of than 1.0. Two undesirable possibilities are that an article is accepted and has an Rw less than 1.0 (Type I error) or is rejected and published elsewhere with an Rw of greater than 1.0 (Type II error). We calculated the distribution (percentage) of all four possibilities. We defined the success rate to be the proportion of articles that were accepted with an Rw>1.0 or rejected with Rw<1.0, divided by all submitted articles.

### Analyses

We explored the relationship between the reviewer ratings of manuscript quality and 1) the reviewer's recommendation, 2) the JGIM publication decision, 3) whether or not the manuscript was eventually published and 4) the impact (Rw) for published articles. We also explored the relationship between JGIM publication decisions with the Rw and compared the impact of articles published in JGIM or rejected by JGIM and subsequently published elsewhere.

We explored these relationships with either the Student's t-test or Analysis of Variance. Correlations were measured using the Pearson correlation coefficient. Internal consistency of the review instrument was assessed with the Cronbach alpha. We assessed agreement using either intraclass correlation coefficients or quadratic kappas. We dichotomized the Rw at 1.0 to create receiver operator curves (ROC) and for logistic regression modeling. We created an ROC curve for the relationship between total quality rating and article impact to examine how well reviewer quality scores “diagnose” high impact articles. We did this by calculating the sensitivity and specificity at each cutpoint for the reviewer total quality rating. We also explored the potential impact on citation rates of three additional scenarios: 1) What would the impact be of varying acceptance rates on citation rates and would there be an optimal acceptance rate? 2) What would the impact be on using strict acceptance for published manuscripts with total quality scores greater than 0 to max total quality score? 3) What would be the impact of basing acceptance rates for published manuscripts on specific subscores? All calculations were performed using STATA (v. 11.2, College Station, Tx).

## Results

### Outcomes of JGIM review process

**Figure 1:**
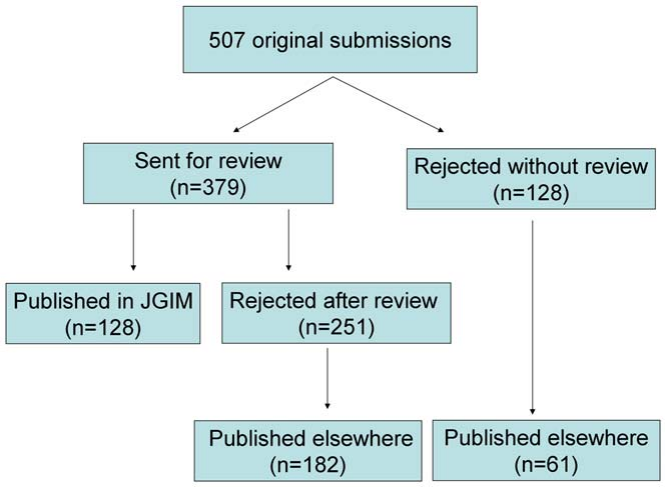
Flowchart of submitted articles.

During the year between 1 June 2004 and 1 July 2005, there were 507 original research articles submitted for the regular JGIM issue. Of these, 128 (25%) were eventually accepted, 331 (65%) were rejected (128 without review), and 48 (25%) were either not resubmitted after revision was requested or were withdrawn by the author after review (Figure 1).

### Reviewer ratings

**Figure 2:**
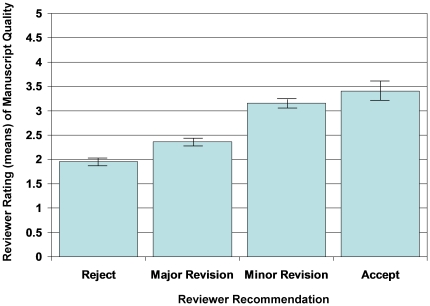
Relationship between reviewer rating of manuscript quality and reviewer recommendation.

All articles published in JGIM were sent for external review. There were a total of 1017 reviewer recommendations for the 379 reviewed articles: 11 (3%) had one, 102 (27%) had two, 262 (69%) had three and 4(1%) had four reviewers. Among these 1017 reviews, the reviewer recommendation was rejection in 285 (28%), reconsider after major revision in 305 (30%), reconsider after minor revision in 223 (22%), conditional accept in 132 (13%) and accept “as is” in 72 (7%) reviews. Reviewers' quality ratings had good internal consistency among the 6 quality domains (Cronbach alpha 0.79). There was a linear correlation between the reviewer publication recommendations (reject, major revision, minor revision, accept) and the average quality rating of the manuscript (β = 0.38, 95% CI: 0.34–0.42, p<0.0005); articles recommended to be rejected had an average quality rating of 1.6, while those recommended for acceptance “as is” averaged 3.40 (Figure 2). Finally, articles published in JGIM had quality ratings that were higher than articles that were rejected (Table 1). However, for a given manuscript there was only modest correlation between reviewer average quality ratings with intraclass correlations ranging from 0.09 to 0.13 and low levels of agreement between reviewers on their recommendations with weighted kappas between the reviewers ranging from 0.11 to 0.15.

**Table 1:** Relationship between ratings by peer reviewers and odds of acceptance.

Quality Domain: Peer review scores	Articles accepted by JGIM	Articles rejected by JGIM		Odds of Acceptance (95% CI)	Rw (β coefficient, 95% CI)
		Published Elsewhere	Unpublished		
Average Total Quality Score, mean?	3.38	2.75	2.48	1.89 (1.49–2.41)	0.20 (0.02–0.37)
Interest to JGIM Readers, mean (0–5)	3.92	3.33	3.39	1.85 (1.54–2.22)	0.11 (–0.01 to 0.23)
Originality, mean (0–5)	3.64	2.99	2.88	1.99 (1.65–2.37)	0.03 (–0.08 to 0.15)
Study Design, mean (0–3)	1.14	1.0	0.33	4.15 (0.54–31.8)	0.03 (–0.20 to 0.26)
Analysis, mean (0–5)	3.18	2.81	2.31	1.55 (1.31–1.82)	0.08 (–0.04 to 0.19)
Validity of Conclusions, mean (0–5)	3.64	2.91	2.81	2.08 (1.73–2.49)	0.13 (0.01–0.25)
Clarity of writing, mean (0–5)	3.80	3.33	3.03	1.56 (1.33–1.82)	0.21 (0.10–0.32)

**Figure 3:**
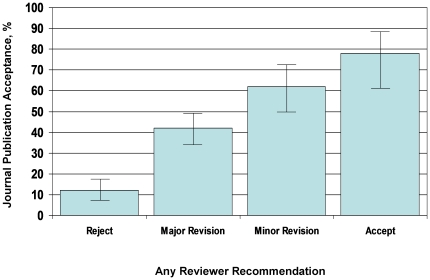
Influence of any reviewer recommendation and journal publication decision.

There was evidence that the editors were influenced by the reviewers' recommendations. If any reviewer recommended reject, this markedly reduced the likelihood of eventual acceptance by JGIM (OR: 0.12, 95% CI: 0.07–0.19); conversely, a recommendation of accept “as is” increased the likelihood of acceptance (OR: 5.23, 95% CI: 2.45–11.21). There was a stepwise increase in the likelihood of acceptance as any reviewer recommended reject, reconsider with major revision, reconsider with minor revision and accept (Figure 3). Most of the quality domains had an impact on the likelihood of acceptance (Table 1). For example, every 1 point increase in the average quality rating of “interest to JGIM readers” increased the odds of acceptance by 1.85 (95% CI: 1.54–2.22). In multivariable models, interest to JGIM readers (OR: 1.33, 95% CI: 1.07–1.66), originality (OR: 1.43, 95% CI: 1.15–1.77) and validity of conclusions (OR: 1.69, 95% CI: 1.39–2.06) independently increased the likelihood of acceptance. Study design, statistical analysis and clarity of writing did not independently contribute to decision-making.

### Article Outcomes

Among all submitted articles, including unpublished articles, the average number of citations over the subsequent 3 years was 5.2 (95% CI: 4.5–5.8).

#### Articles accepted by JGIM

**Figure 4:**
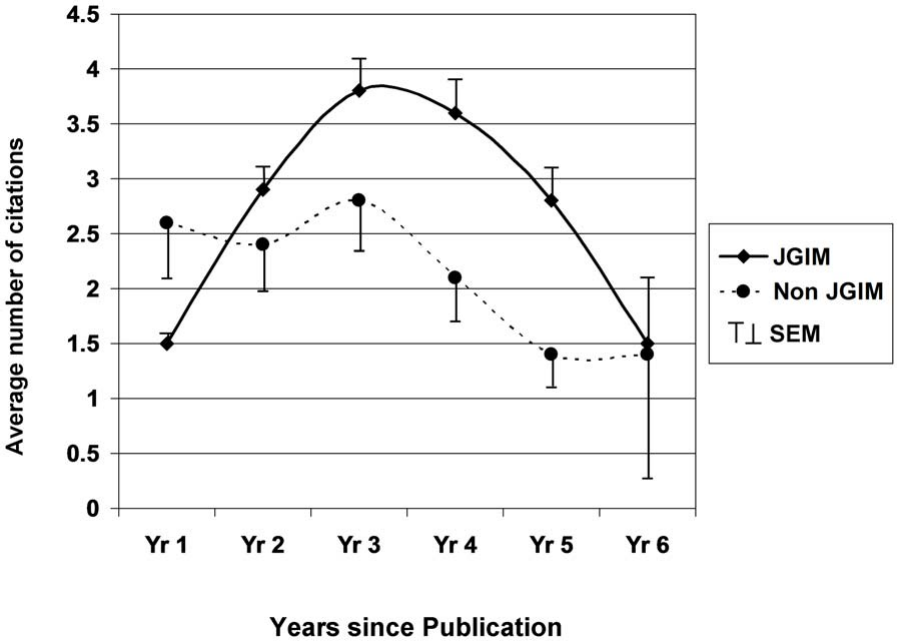
Years since publication and citation rate.

Among the 128 articles accepted by JGIM, 83 (65%) were published in 2005, 44 (34%) in 2006 and one in 2007. For the 3 years immediately after publication, the average number of citations was 8.1 (95% CI: 6.8–9.3). When followed for up to 6 years, the peak number of citations occurred in the third year after publication (Figure 4). The mean Rw for all JGIM published articles was 1.6 (95% CI: 1.3–1.8), with 73 (54%) having an Rw greater than expected for this cohort of articles.

#### Articles rejected by JGIM

Among the 331 articles rejected by JGIM, 243 (73%) were eventually published by 84 different journals. Articles that were sent out for review but eventually rejected were more likely to eventually be published than those rejected without review (RR: 1.50, 95% CI: 1.04–2.14). Among rejected articles that were eventually published elsewhere, 5 (2%) were published in 2004, 76 (29%) in 2005, 120 (46%) in 2006, 18 in 2007 (7%), 19 (7%) in 2008 and five (2%) in 2009. There was an average 8.8 month delay (95% CI: 5.9–11.5 months) in publication between articles accepted and published in JGIM and manuscripts rejected by JGIM and published elsewhere. Articles published elsewhere had an average number of citations of 5.7 (95% CI: 4.7–6.7) with a mean Rw of 1.10 (95% CI: 0.91–1.29).

### Reviewer Rating and Article Importance

**Figure 5:**
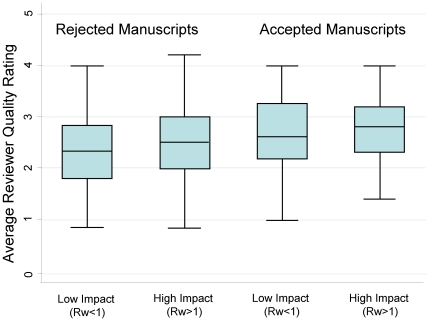
Quality review ratings and Impact of Article.

There was evidence of a relationship between average quality ratings and final outcome of submission, with a stepwise increase in rating between unpublished articles, articles rejected but eventually published in another journal and those published in JGIM (Table 1). There was also evidence of a relationship between the average quality rating and the article impact (Rw, Table 1). Among the six quality domains, there was a significant relationship between “validity of conclusions” and “clarity of writing” and the Rw (Table 1). For example, for every one point increase in the average quality rating, the Rw increased by 0.20 (95% CI: 0.02–0.37). However, the standardized effect size (ES) for each of these domains was small (average quality: ES: 0.10; validity: ES: 0.10; clarity of writing: ES: 0.17), suggesting that the effect was weak [16]. Moreover, a receiver operator curve (ROC) demonstrates that the average reviewer quality rating does a poor job of distinguishing articles destined to have higher or lower than average impact with an area under the curve of 0.59. There appeared to be no quality cutpoint that accurately distinguished higher from lower impact articles (Figure 5). However, there was an increase in the proportion of submissions correctly classified (accepted with an Rw>1 or rejected with an Rw<1) as the number of reviewers increased from 2 reviewers (35%) to 3 reviewers (69%).

### Comparison between accepted and rejected articles

#### Citation rate

**Figure 6:**
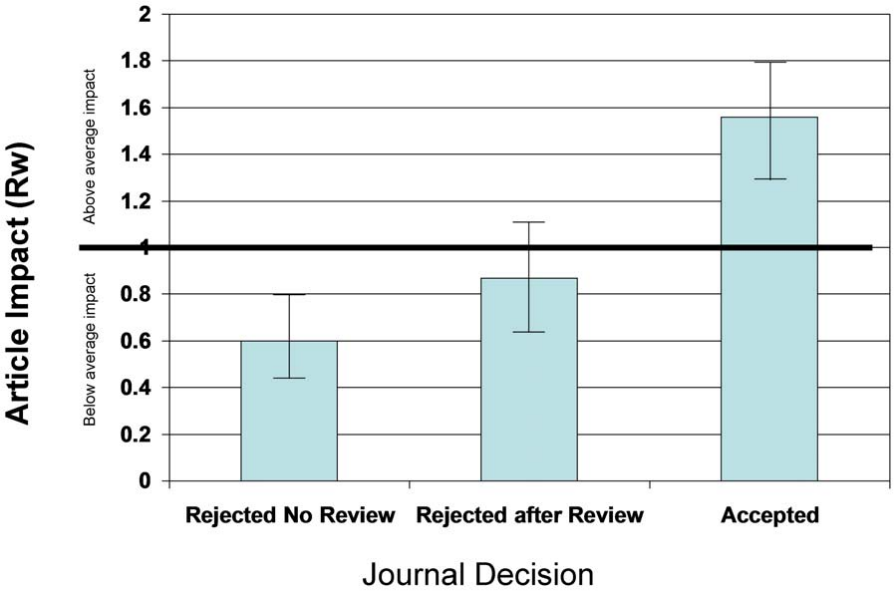
Relationship between editor decision and article impact.

Articles eventually published in another journal had a lower average Rw than those published in JGIM (1.6 vs. 1.1, p = 0.002). There was a stepwise increase in Rw between articles that were rejected without review (Rw: 0.60), rejected after review (Rw: 0.87) and accepted (Rw: 1.56, p<0.0005 for difference between groups, Figure 6).

#### Accuracy of JGIM Decision

Seventy three (14%) of all submissions accepted by JGIM and had an Rw greater than expected; 287 (57%) of all submissions were rejected and were either unpublished or had an Rw less than expected for JGIM. Hence, 71% of decisions resulted in desirable outcomes from the journal's point of view. However, 55 (11%) of articles were accepted and had lower than expected citation rates (Type I error) and 92 (18%) were rejected and subsequently had higher than average citation rates (Type II error). Hence undesirable reviewer outcomes occurred in 29% of submissions.

### Alternative Selection Methods

#### Random selection

Over a range of random selection rates from 1% to 100%, there was no significant difference in any of the randomly selected samples from the mean of the group (Rw: 1.0). The average for the entire range was 1.0 (95% CI: 0.73–1.29). This should not be particularly surprising since a correctly performed random sample should provide average results that reflect the characteristics of the population sampled. A method of selecting articles randomly would thus only result in publishing articles that reflected the potential citation rate of the group of articles originally submitted and would fail to adequately distinguish high from low impact articles.

#### Absolute quality scores

**Figure 7:**
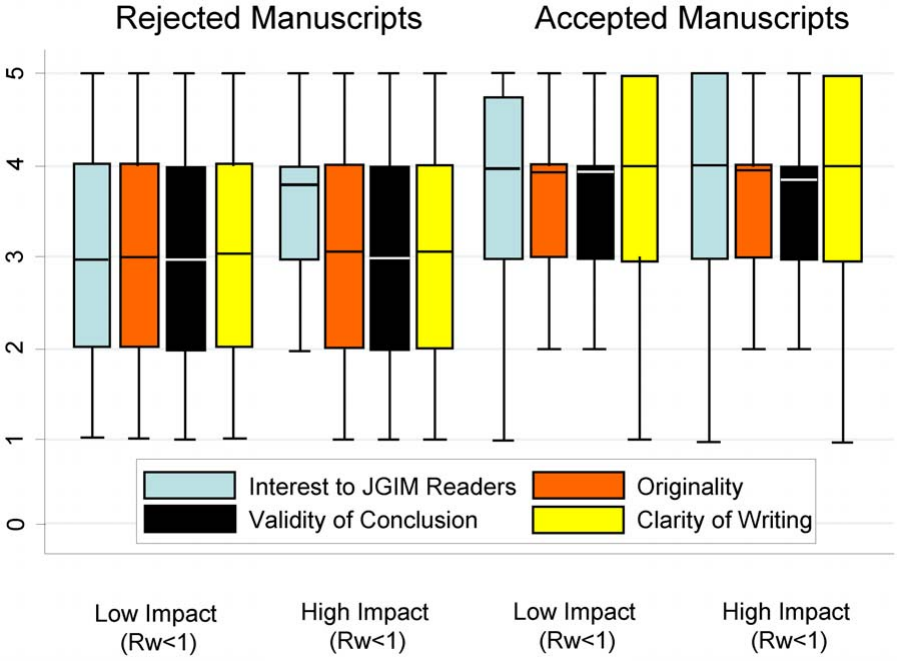
Reviewer domain quality scores and article impact.

Graphs of the average quality (Figure 5) and scores for specific domains, interest to JGIM readers, paper originality, validity of conclusions and clarity of writing, (Figure 7) revealed no cut-point that would differentiate between high and low impact articles.

## Discussion

We found evidence that biomedical journal peer review largely succeeds in selecting high impact articles for publication and dispatching lower impact articles, but the process is far from perfect. While 71% are correctly classified, 29% are not, with some accepted articles having lower than average impact, and some rejected articles having higher than average impact. This rate of successful sorting of submissions is nearly identical to that seen in the study of peer review in a high impact chemistry journal [11]. We found that raters had good internal consistency in the ratings they gave in the 6 quality domains and good agreement between these ratings and their recommendation regarding publication, but low inter-rater reliability. This is similar to findings from previous studies [7]–[10]. However, we found that the editor decisions regarding publication were fairly accurate in discriminating high from low impact articles.

There are several possible explanations for this finding. First, editors commonly solicit reviewers with different background and perspectives. For example, an article using qualitative methods about patient-doctor communication may prompt the editor to obtain a review from a reviewer with expertise in qualitative methods and another from an expert in patient-doctor communication. It may not be surprising that two experts looking at the same paper from different perspectives may rate the articles differently and make divergent recommendations. Secondly, we had no assessment of the quality of each of the reviews. Review quality varies widely from reviewer to reviewer. This could contribute to lack of agreement. It is uncertain whether two highly rated reviewers would have better agreement rates. One study found that there was low agreement between the editor's decision and reviewer recommendations regarding publication, even among reviews that were rated as high quality [6]. It is also possible that different reviewers value some article traits more highly than others: some may emphasize clarity of writing, others the timeliness or originality of the material. It may not be surprising that we, like others, have found low inter-rater agreement among the reviewers of scientific articles.

What is notable is that from this morass of conflicting advice comes a decision that fairly accurately discriminates high from low quality articles. While editors are clearly being influenced by reviewer's recommendations, they appear to synthesize the comments and ratings and arrive at decisions that are more accurate than would be suggested by the low relationship between individual reviewer quality ratings or recommendations and article impact.

There are a number of limitations to our study. First, an alternative explanation for the internal consistency of reviewer ratings is a halo effect, in which a rater might tend to assign the same number for all quality domains assessed. While this could partially explain the Cronbach alpha for the six quality domains, it would not explain the consistency of the relationship between quality ratings and the specific recommendation made. Secondly, an alternative explanation for the finding that rejected articles have lower impact is that there is a natural selection that occurs as authors decide where to submit their articles. The typical submission pattern is for authors to submit first to higher then to lower impact journals. While it is likely this bias contributes to our findings, this is probably not as strong a factor for a journal like the Journal of General Internal Medicine, with a modest impact factor than it would be for a more highly rated journal. A second explanation for the incremental increase in citation rates between articles rejected without review and those rejected after review is that the authors in their submission to another journal incorporated the advice they received from the JGIM reviewers and editors. While it is possible that this attenuates some of the difference between rejected with and without review citation rates, it is unlikely to explain the entire difference. Moreover, if such an effect existed, it would tend to reduce the difference we found between those articles published in JGIM and those that were reviewed but published in a journal other than JGIM.

We also found evidence that 3 reviewers are better than 2 as the percent of submissions correctly classified increased from 35% to 69%. It is impossible to determine from our data the optimal number of reviews. It is also uncertain whether the extra costs associated with obtaining additional reviews would be worthwhile since the editor's decisions appear to reasonably discriminate high from low impact articles.

There is interest in using absolute cut points of quality scores to make decisions about accepting or rejecting articles. Our data suggests that making editorial decisions based on total quality scores or the score on a specific quality domain would not adequately discriminate between high and low impact articles, as nicely demonstrated in the ROC curve.

Like most journals, the JGIM peer review process has an element of subjectivity. While the deputy editors undergo some training to standardize the process of decision-making, external peer reviewers are volunteers. They are given limited written instructions and may access the JGIM website for further guidance or attend an annual workshop for reviewers but are not required to undergo training before submitting reviews. External peer reviewers are asked to self-select their interests and expertise and this information is used in selecting reviewers for articles. Reviewers may have personal biases for or against particular types of research that may influence their recommendation and may possess varying degrees of knowledge in the area. In addition, the decision to accept an article includes other factors that may not be fully captured by our data, such as timeliness or importance of the topic to the journal's parent organization, the Society of General Internal Medicine.

Despite these limitations, peer review appears to be useful. Article selection by journals based on peer review may be important as journals compete for higher impact ratings, as measured by the ISI citation index. A journal's calculated ISI score affects journal prestige, influences authors' decisions about where to submit their best work, and may affect advertising revenue. It was also identified by the Cochrane collaboration as the best surrogate marker for article importance [14]. However, the ISI impact factor measures just one aspect of article quality – the extent to which other researchers cite the manuscript. It does not capture how often the information is read (let alone used) by practitioners, read by the public, disseminated in the media, or used to make policy decisions. (Suitable surrogate metrics for these outcomes might include eigenfactors, article downloads, websearches, mentions in the popular press, or citations in public speeches, respectively.) Additionally, article type can affect impact. Important health policy topics have a shorter half-life of interest, and may have lower citations. Medical education topics have a relatively narrow audience (primarily medical educators), even when well done and useful. Thus, the proportion of article topics within a journal will profoundly affect a journal's perceived value, even with rigorous peer review. It is thus not surprising that studies that use the citation index as the only measure of “usefulness” of an article may find only weak correlations with the final decision or with individual rater recommendations.

In summary, this study shows that peer review in combination with editorial judgment at JGIM is reasonably good at picking future “winners”. While the individual reviewers have good consistency, they have low agreement. There also does not appear to be a particular quality cut point that will discriminate high from low impact articles. Journal editors take these often conflicting recommendations into account and appear to synthesize them in reaching publication decisions. It also appears that a larger number of reviewers is better, though the ideal number cannot be determined from our data. Nevertheless the process could be improved, especially with respect to the hidden gems that are rejected by JGIM and then go on to garner many citations. While JGIM is not alone in its imperfections (Nature initially rejected Stephen Hawking's paper on black hole radiation), more work is needed to improve the reliability and validity of the peer review process. Wrong decisions are inevitable; fortunately there are numerous opportunities for authors to publish medical articles. Hawking did eventually publish his seminal work. It is likely that worthy articles eventually find a place in the published literature.

## References

[1] Pierie J, Walvoort HC, Overbeke AJPM (1996). Readers' evaluation of effect of peer review and editing on quality of articles in the Nederlands Tijdschrift voor Geneeskunde.. Lancet.

[2] Goodman SN, Berlin J, Fletcher SW, Fletcher RH (1994). Manuscript quality before and after peer review and editing at Annals of Internal Medicine..

[3] Arnau C, Cobo E, Ribera JM, Cardellach F, Selva A (2003). Efecto de la revision estadistìca en la calidad de los manuscritos publicados en Medicina Clìnica: estudio aleatorizado.. Med Clin (Barc).

[4] Day FC, Schriger DL, Todd C, Wears RL (2002). The use of dedicated methodology and statistical reviewers for peer review: a content analysis of comments to authors made by methodology and regular reviewers.. Annals of Emergency Medicine.

[5] Shattell MM, Chinn P, Thomas SP, Cowling WR (2010). Authors’ and editors’ perspectives on peer review quality in three scholarly nursing journals.. J of Nursing Scholarship.

[6] Weller AC (1996). Editorial peer review: A comparison of authors publishing in two groups of U.S. medical journals.. Bulletin of the Medical Library Association.

[7] Lock, Stephen (1985). A Difficult Balance: Editorial Peer Review in Medicine..

[8] Kravitz RL, Franks P, Feldman MD, Gerrity M, Byrne C (2010). Editorial peer reviewers' recommendations at a general medical journal: are they reliable and do editors care?. PLoS One.

[9] Marusic A, Mestrovic T, Petrovecki M, Marusic M (1998). Peer review in the Croatian Medical Journal from 1992 to 1996.. Croat Med J.

[10] Gupta P, Kaur G, Sharma B, Shah D, Choudhury P (2006). What is submitted and what gets accepted in Indian Pediatrics: analysis of submissions, review process, decision making, and criteria for rejection.. Indian Pediatr.

[11] Callaham ML, Baxt WG, Waeckerle JF, Wears RL (1998). Reliability of editors' subjective quality ratings of peer reviews of manuscripts.. JAMA.

[12] Bormann L, Daniel HD (2010). The usefulness of peer review for selecting manuscripts for publication: A utility analysis taking as an example a high-impact journal.. PLoS One.

[13] Bornmann L, Daniel HD (2009). Extent of type I and type II errors in editorial decisions: a case study on Angewandte Chemie International Edition.. Journal of Informetrics.

[14] Jefferson T, Rudin M, Brodney-Folse S, Davidoff F (2008). Editorial peer review for improving the quality of reports of biomedical studies. Cochrane Database of Systematic Reviews, Art. No.. MR.

[15] Vinkler P (1997). Relations of relative scientometric impact indicators. The relative publication strategy index.. Scientometrics.

[16] Kazis LE, Anderson JJ, Meenan RF (1989). Effect sizes for interpreting changes in health status.. Med Care.

